# Supplementary material for: Identification of an Embryonic Cell-Specific Region within the Pineapple SERK1 Promoter
Source: Genes (Basel). 2019 Nov 1;10(11):883. doi: 10.3390/genes10110883 (PMC6896011; doi:10.3390/genes10110883)
Supplement: Supplementary file 1 [file genes-10-00883-s001.zip › Figure S2.pdf]

Identification of an Embryonic Cell-Specific Region within the Pineapple *SERK1* Promoter

***Aiping Luan*[1,2], *Junhu He*[1], *Yehua He*[2,*], *Tao Xie*[2], *Chengjie Chen*[2], *Qi Mao*[2], *Xiaoshuang Wang*[2], *Chuhao Li*[2], *Yaqi Ding*[2], *Wenqiu Lin*[2], *Chaoyang Liu*[2] and *Jingxian Xia*[2]**

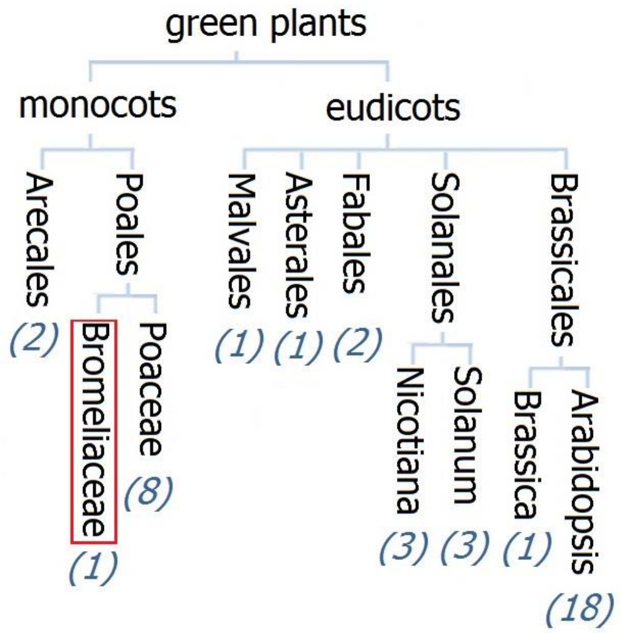

Figure S2. 40 *SERK* family genes associated with somatic embryogenesis in different green plants.
