# Supplementary material for: Identification of an Embryonic Cell-Specific Region within the Pineapple SERK1 Promoter
Source: Genes (Basel). 2019 Nov 1;10(11):883. doi: 10.3390/genes10110883 (PMC6896011; doi:10.3390/genes10110883)
Supplement: Supplementary file 1 [file genes-10-00883-s001.zip › Figure S1.pdf]

Identification of an Embryonic Cell-Specific Region within the Pineapple *SERK1* Promoter

Aiping Luan[1,2], Junhu He[1], Yehua He[2,], Tao Xie[2], Chengjie Chen[2], Qi Mao[2], Xiaoshuang Wang[2], Chuhao Li[2], Yaqi Ding[2], Wenqiu Lin[2], Chaoyang Liu[2] and Jingxian Xia[2]*

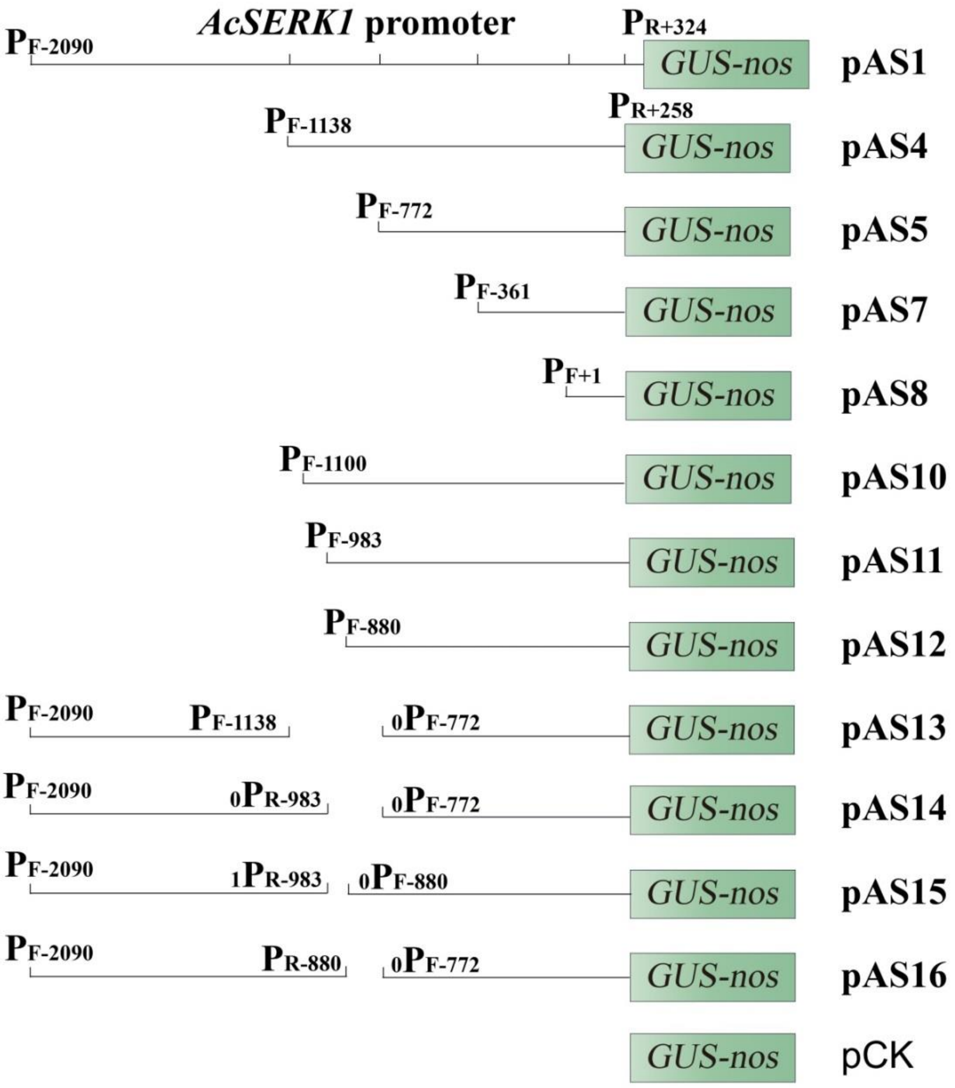

Figure S1. Structure of *AcSERK1* 5' upstream regulatory sequence deletion vectors.
