# Supplementary figures and images for: Identification of an Embryonic Cell-Specific Region within the Pineapple SERK1 Promoter
Source: Genes (Basel). 2019 Nov 1;10(11):883. doi: 10.3390/genes10110883 (PMC6896011; doi:10.3390/genes10110883)

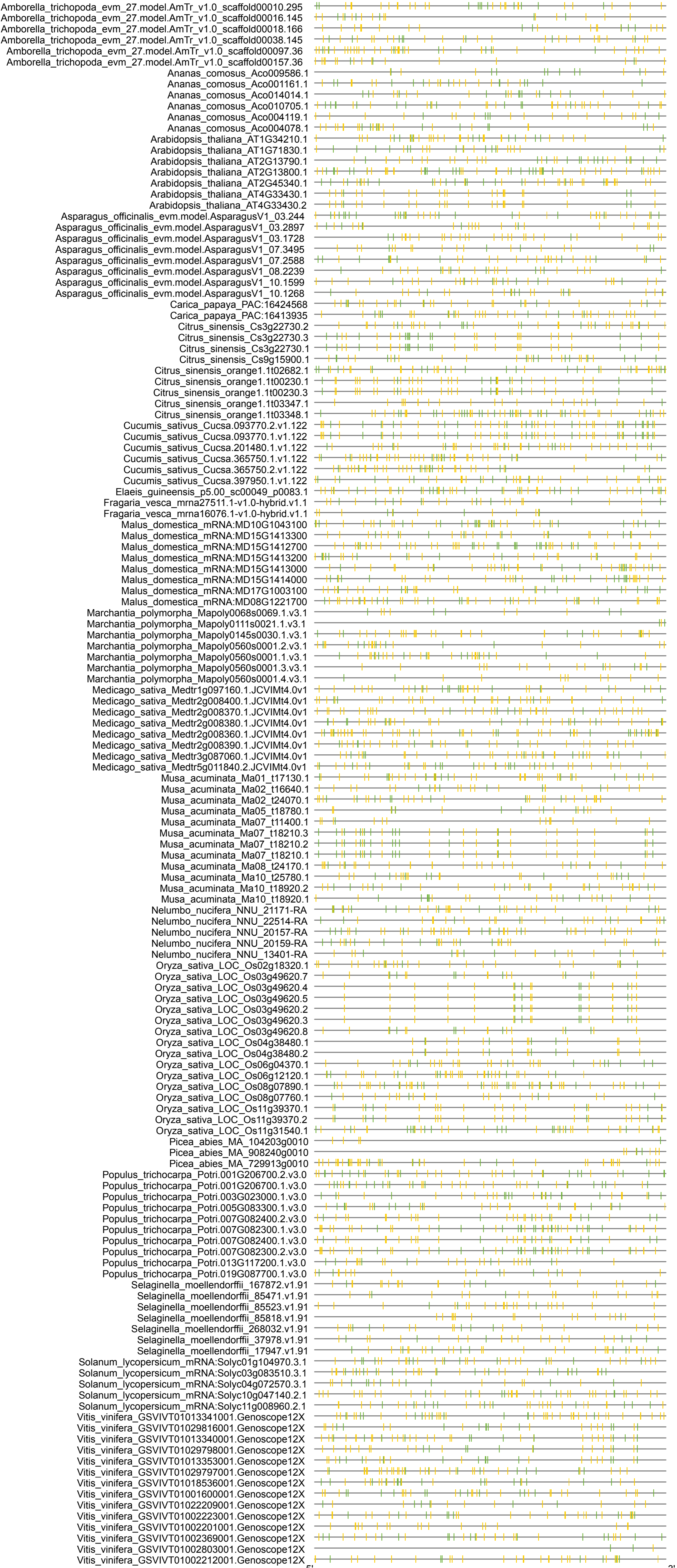

Supplement: Supplementary file 1 [file genes-10-00883-s001.zip › Figure S3.pdf]
